# A multi-stakeholder approach to the co-production of the research agenda for medicines optimisation

**DOI:** 10.1186/s12913-021-06056-5

**Published:** 2021-01-13

**Authors:** John Fellenor, Nicky Britten, Molly Courtenay, Rupert A. Payne, Jose Valderas, Rachel Denholm, Polly Duncan, Deborah McCahon, Lynn Tatnell, Richard Fitzgerald, Krystal Warmoth, David Gillespie, Katrina Turner, Margaret Watson

**Affiliations:** 1 Department of Pharmacy & Pharmacology, University of Bath, Bath, England; 2 University of Exeter Medical School, University of Exeter, Exeter, England; 3 School of Healthcare Sciences, Cardiff University, Cardiff, Wales; 4 Centre for Academic Primary Care, Bristol Medical School, University of Bristol, Bristol, England; 5 Health Services & Policy Research Group, Collaboration for Academic Primary Care (APEx), University of Exeter, Exeter, England; 6 Peninsula Public Involvement Group, University of Exeter, Exeter, England; 7 Centre for Trials Research, College of Biomedical & Life Sciences, Cardiff University, Cardiff, Wales; 8 Strathclyde Institute of Pharmacy and Biomedical Sciences, University of Strathclyde, 161 Cathedral Street, Glasgow, G4 0RE Scotland

**Keywords:** Medicines optimisation, Polypharmacy, Deprescribing, Patient concerns, Non-medical prescribing, Nominal group technique

## Abstract

**Background:**

Up to 50% of medicines are not used as intended, resulting in poor health and economic outcomes. Medicines optimisation is ‘a person-centred approach to safe and effective medicines use, to ensure people obtain the best possible outcomes from their medicines’. The purpose of this exercise was to co-produce a prioritised research agenda for medicines optimisation using a multi-stakeholder (patient, researcher, public and health professionals) approach.

**Methods:**

A three-stage, multiple method process was used including: generation of preliminary research questions (Stage 1) using a modified Nominal Group Technique; electronic consultation and ranking with a wider multi-stakeholder group (Stage 2); a face-to-face, one-day consensus meeting involving representatives from all stakeholder groups (Stage 3).

**Results:**

In total, 92 research questions were identified during Stages 1 and 2 and ranked in order of priority during stage 3. Questions were categorised into four areas: ‘Patient Concerns’ [e.g. is there a shared decision (with patients) about using each medicine?], ‘Polypharmacy’ [e.g. how to design health services to cope with the challenge of multiple medicines use?], ‘Non-Medical Prescribing’ [e.g. how can the contribution of non-medical prescribers be optimised in primary care?], and ‘Deprescribing’ [e.g. what support is needed by prescribers to deprescribe?]. A significant number of the 92 questions were generated by Patient and Public Involvement representatives, which demonstrates the importance of including this stakeholder group when identifying research priorities.

**Conclusions:**

A wide range of research questions was generated reflecting concerns which affect patients, practitioners, the health service, as well the ethical and philosophical aspects of the prescribing and deprescribing of medicines. These questions should be used to set future research agendas and funding commissions.

**Supplementary Information:**

The online version contains supplementary material available at 10.1186/s12913-021-06056-5.

## Background

Globally, medicines are the most commonly used healthcare intervention [1]. In the United Kingdom (UK), for example, over 1 billion prescription items are dispensed annually in the community, many for chronic health conditions [2]. Up to 50% of medicines are not used as intended [3] and, as a result, health outcomes are sub-optimal. Both over- and under- use of medicines leads to diminished benefits, greater costs and increased harms [3], and is viewed by the World Health Organisation (WHO) as a global patient safety problem [4].

Medicines optimisation, ‘a person-centred approach to safe and effective medicines use, to ensure people obtain the best possible outcomes from their medicines’ [5], focuses on ensuring that the patient derives the most benefit from their medicines, and requires a holistic approach and an effective partnership between health professionals and patients [5, 6]. It also includes deprescribing, i.e. the process of withdrawing a patient’s medicine to improve health or mitigate against possible adverse side effects [7, 8]. Medicines optimisation is of relevance and importance to a wide range of stakeholders including patients, the public, healthcare professionals, health service commissioners and policymakers. Achieving the optimal use of medicines is complex due to competing priorities and agendas of different stakeholders [9]. The development of relevant policy requires an expansion of the evidence base, which reflects the needs of all stakeholders. This is apposite as medicines optimisation is typically under-represented in current models of care [10].

Involving patients and their advocates in the co-production of health services and research is becoming more commonplace [11]. Co-production leads to differentiated services and choice, increased responsiveness to changing needs, and reduced waste and costs [12]. It emphasises the contribution that all stakeholders can make as initiators *or* recipients of the service delivery process [12, 13] and is based on egalitarian relationships between experts and lay people, using a process of open exchange and participation [14]. The inclusion of the public and other stakeholders in research agenda setting is increasing [15–17] but until now, has not included medicines optimisation. The aim of this exercise was to adopt a multi-stakeholder approach to the co-production of a prioritised research agenda for medicines optimisation. In so doing, the agenda could be used to inform future funding initiatives and activities. This process was undertaken as a GW4 Alliance [18] research initiative; a collaboration between the Universities of Bath, Bristol, Cardiff and Exeter.

## Method

### Study design

The prioritisation process involved multiple stages and methods. These included identification of a broad range of stakeholders, face-to-face meetings to generate initial research questions, and a one-day workshop to prioritise the research questions generated. The overall process was based on a modified Nominal Group Technique (NGT) [19–21]. The NGT is usually conducted with homogenous groups [22].

### Stage 1: generation of preliminary research questions

The first stage involved the identification of key stakeholders within the GW4 Alliance institutions, e.g. academics with expertise in pharmacy and pharmacology, medical and other healthcare professionals, and patient and public involvement representatives i.e. health service users and organisational representatives. In August 2018, a face-to-face stakeholder meeting was convened (by the corresponding author), to undertake a modified Nominal Group Technique (NGT) [19–21]. Participants were provided background information and asked to address thequestion: ‘*What are* the *priority topics/areas that need to be addressed so that medicines optimisation can be realised?’.* During the meeting, participants, including the research team, were encouraged to generate as many questions as possible in response to the research question; these were recorded as individual written responses and collated on flip-charts. No discussion was permitted until the generation process was complete. Discussion then followed for the purpose of clarification of questions, removal of duplicates and the identification of common themes. Following the meeting, the questions were reviewed by the research team and refined to produce a distilled list of research questions for consideration in Stage 2. Each question was assigned to one of four categories that reflected common themes: ‘patient concerns’, ‘polypharmacy’, ‘non-medical prescribing’ (NMP), and ‘deprescribing.

### Stage 2: consultation with wider stakeholder group

The purpose of Stage 2 was to seek input from a wider stakeholder group regarding the original research questions identified in Stage 1. In addition to Stage 1 participants, an email invitation was sent to 80 individuals identified from relevant literature and policy documents and via the professional networks of the core research team and which included a wide range of local, regional, national, and international stakeholders (e.g. pharmacists, academic pharmacists, physicians, National Health Service (NHS) Trust directors, patients, physicians, health workers, and advocacy organisations (including Age UK and the Patients’ Association)). All questions from Stage 1 were presented in a document, using the four categories, and emailed to all prospective participants. The task for participants was to rank the Stage 1 questions according to their perceived importance, and to add new questions from their own ideas/experiences. For each original question, a mean rank was calculated using the Excel rank function. Additional questions suggested by participants were sense checked with duplicate questions removed or combined and then assigned to one of the original four question categories according to its content.

### Stage 3: final prioritisation

Stage 3 comprised a one-day prioritisation workshop in November 2018, held on University of Bath campus and facilitated the lead author (MCW). All respondents in Stages 1 and 2 were invited. Participants and the research team were purposively assigned to one of four groups comprising eight individuals, to ensure that each group included a range of participants, e.g. at least one lay representative, and representatives from each stakeholder group.

The research questions in each of the four categories were discussed within each group with each category being assigned 45-min discussion session. Discussion included the opportunity to reflect on the mean rank of questions from Stage 1.

Following discussion session, the participants rated the importance of each question. TurningPoint software was used, which facilitates live polling and as well as the curation and simple statistical analysis of results [23]. Each question was presented alongside a Likert scale, ranging from one (‘extremely important’), to seven (‘extremely unimportant’). Participants rated each question independently. The process was then repeated for three remaining categories of research questions.

Following the meeting, the questions were presented in ordered rank to derive a definitive list of prioritised research questions (Additional file 1; a list of all 92 questions alongside their rank, how they were rated by PPIs and non-PPIs, and the percentage of ‘extremely important’ and ‘important’ ratings).

Patient and public participants received a participation fee and their travel expenses were reimbursed. Non-PPI participants had their travel expenses reimbursed if requested, but received no additional payment for their involvement.

#### Ethical approval and consent

Ethical approval was not required for this study (confirmed by the Ethics Officer of the Department of Pharmacy and Pharmacology, University of Bath). As such, signed consent was not sought from any participant. Participation in, and completion of, each stage was accepted as participants’ consent to participate.

### Data analysis

A Borda count [24, 25] was used to rank the order of questions prioritised in Stage 3, where the Likert rating ‘extremely important’ was given a weighting of 7, ‘important’ a weighting of 6 and so forth, to ‘extremely unimportant’ weighted as 1. Following weighting, the number of times a question was rated as ‘extremely important’ was combined with the number of times it was rated as ‘important’ etc. For example, if 19 people rated a question as ‘extremely important’, eight rated it as ‘important’, and five rated it ‘somewhat important’, then its overall weighted score would be (19 × 7) + (8 × 6) + (5 × 5) = 206. Weighted question totals were subsequently ranked according to median score.

Using the process described above, the top five questions prioritised in each topic area by PPI participants were compared with the rank given to the selected questions by all other participants (designated as non-PPI). The purpose of this comparison was to determine whether substantial differences existed in the type of question that both types of participants prioritised. Such information provides greater insight into the heterogeneity of different stakeholder types. During ranking exercises, participants often rank their most and least favourite choices, based for example on familiarity with concepts, therefore middle rankings may reflect more arbitrary or indifferent choices [26]. As such, the five highest ranked questions are discussed as this number will likely capture the broad range of what participants have actively considered as most important, while allowing sufficient coverage of the overall question set.

## Results

### Stage 1: generation of preliminary research questions

**Fig. 1:**
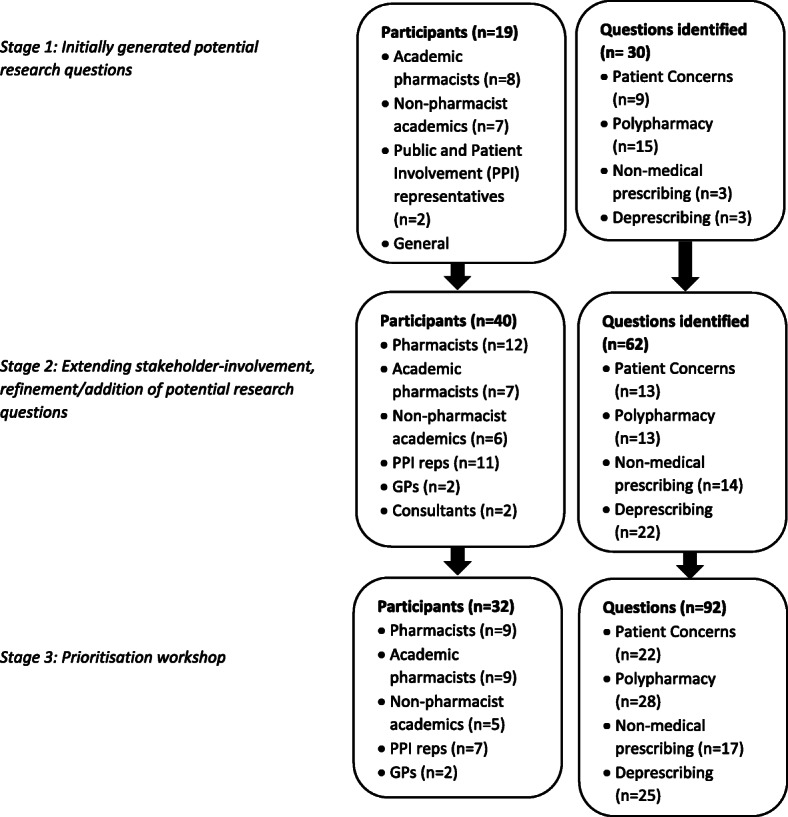
The identification and prioritisation of medicines optimisation research questions: numbers of participants and questions generated by stage

Nineteen individuals participated. Of these, the majority were academics, eight of whom had a pharmacy background (Fig. 1), as well as two GPs and two PPI representatives. Thirty questions were generated and grouped into four categories. All five questions pertaining to patient concerns were generated by PPI representatives.

### Stage 2: consultation with wider stakeholder group

Forty stakeholders, including those from Stage 1, participated in Stage 2 (Fig. 1); two Stage 1 participants did not participate in any later stages of this process. Approximately one third of participants were academics and four participants had a medical background. In total, 62 new questions were generated across the four categories: patient concerns (13), polypharmacy (13), non-medical prescribing (14), and deprescribing (22). Of these, 28 were generated by PPI representatives. All the additional questions nominally reflected the four categories. Four suggestions were omitted due to ambiguity of meaning e.g. “Need to do more about process”. Some questions, reflected more than one category. For example, “How is deprescribing, polypharmacy, etc undertaken by NMPs?”. These questions were assigned to the category deemed most relevant to their content.

### Stage 3: final prioritisation

In total, 32 of the 40 Stage 2 participants participated in the Stage 3 meeting, comprising academic pharmacists (*n* = 9), pharmacists including a regional deputy director of the medicines information service and an NHS Trust regional director of pharmacy (*n* = 9), PPI representatives (*n* = 7), academic researchers (*n* = 5), and GPs (*n* = 2).

Over half of the participants provided a rating of ‘extremely important’ or ‘important’ for 13 of 22 patient concerns questions, 14 of 28 polypharmacy questions, 7 of 17 non-medical prescriber questions, and 15 of 25 deprescribing questions. Two questions from the polypharmacy category, relating to the status of theory and a specific intervention tool, received a rating of ‘unimportant’ or ‘extremely unimportant’ from just under a third of participants. No other question in any category received comparably negative ratings. In all categories, ratings were skewed toward the positive end of the scale; only one question in the category of polypharmacy demonstrated polarisation, i.e. equal numbers of positive and negative ratings. This question pertained to whether clusters of co-morbidities are ‘more important’ than the total number of co-morbidities.

Of the top 20 items identified across the four categories (i.e. five per category) 10 had been identified by the wider stakeholder group during Stage 2; five questions received an ‘extremely important’ or ‘important’ rating from at least three-quarters of participants (Table 1). All Stage 3 questions are presented in Additional file 1.

**Table 1:** Top-five ranked questions by topic area

Topic area	Overall rank	Question	PPI rank (***n*** = 7)	Non-PPI rank (***n*** = 25)
Patient Concerns (PC)	1	To what extent are patients’ views and experiences considered during medication reviews?	3	1
	2	*Is there a shared decision (with patients) about using each medicine?	1	2
	3	How are medication reviews by different healthcare professionals integrated?	2	5
	4	How can communication relevant to medication reviews be enhanced across and within sectors?	5	3
	5	What information/education would patients require regarding polypharmacy to engage and take some ownership?	7	6
Polypharmacy (P)	1	*To what extent should polypharmacy involve patients’ views and preferences in shared decision making?	1	5
	2	*If we undertake a polypharmacy medication review well with the right person at the right time, what happens to the patient (i.e. *are their outcomes better, does their quality of life (QoL) improve* etc.*)?*	5	2
	3	*How can we reengineer primary care to make time for medication review to be done well by the right people (since this is only going to get bigger i.e. *more older people on more drugs)?*	8	2
	4	*How do we achieve a “culture shift” to prevent polypharmacy (including engagement with public, addressing socioeconomic disparities)?*	13	1
	5	How should health services be redesigned to cope with polypharmacy/multimorbidity?	11	2
Non-medical Prescribing (NMP)	1	How can we optimise the contribution of non-medical prescribers to primary care?	1	1
	2	How is deprescribing, polypharmacy, etc. undertaken by non-medical prescribers?	5	2
	3	*What are the influences on deprescribing behaviour of non-medical prescribers?*	7	3
	4a	*To what extent does NMP training provide HCPs with the confidence to address complex polypharmacy and deprescribing issues with patients; address inappropriate polypharmacy?*	9	4
	4b	What is the role of the NMP in medicines reviews (e.g. in specialist areas such as rheumatology and type of NMP e.g. pharmacist, nurse)?	3	6
Deprescribing (D)	1	*How can we empower patients to take a more active role in self-management and self-monitoring of multiple long-term conditions, including deprescribing?	1	1
	2	*To what extent is shared decision-making incorporated into deprescribing consultations?*	19	1
	3	*Where are the gaps in education and training (about deprescribing) and how these can be addressed is key to ensuring deprescribing is safe and effective?*	7	3
	4	Should all ‘prescribing’ guidelines include recommendations to deprescribe, and if so how?	2	9
	5	What should the core outcome set be for deprescribing interventions?	9	4

Italicised items represent additional questions suggested by stakeholders during Stage 2; ranks with suffix ‘a’, ‘b’ represent equivalent median scores. Questions receiving an ‘extremely important’ or ‘important’ rating from at least three-quarters of participants are indicated with *

The comparison of PPI versus non-PPI results demonstrates variation across the different types of participants (Table 2). Only nine of the 20 highest ranked questions by PPI participants were ranked highly by the non-PPI participants.

**Table 2:** Top 5 questions by topic and PPI rank, compared with non-PPI rank

**Patient Concerns**	**PPI rank** **(*n*** **= 7)**	**Non-PPI rank** **(*n*** **= 25)**
Is there a shared decision (with patients) about using each medicine?	1	2
How are medication reviews by different healthcare professionals integrated?	2	5
To what extent are patients’ views and experiences considered during medication reviews?	3a	1
What are patients’ perspectives and experiences of non-medical prescribers, and does this vary according to different professional groups and different patient groups?	3b	10
How can communication relevant to medication reviews be enhanced across and within sectors?	5	3
**Polypharmacy**	**PPI rank**	**Non-PPI rank**
To what extent should polypharmacy involve patients’ views and preferences in shared decision-making?	1	5
What can patients teach health care professionals about the burden of polypharmacy and coping (or not) mechanisms?	2	11
Which patients should be targeted by polypharmacy medication reviews, where and when?	3	7
What are the key components of a good, person-centred, holistic, polypharmacy medication review?	4	6
If we undertake a polypharmacy medication review well with the right person at the right time, what happens to the patient (i.e. are their outcomes better, does their ‘quality of life’ improve etc.)?	5	2
**Non-medical Prescribing**	**PPI rank**	**Non-PPI rank**
How can we optimise the contribution of non-medical prescribers to primary care?	1	1
How can we raise patient awareness of these ‘new’ prescribers?	2	10
What is the role of the NMP in medicines reviews (e.g. in specialist areas such as rheumatology and type of NMP e.g. pharmacist, nurse)?	3a	6
How can non-medical prescribers optimise medicines use in vulnerable patient populations e.g. drug misusers, individuals with mental illness?	3b	13
How is deprescribing, polypharmacy, etc. undertaken by non-medical prescribers?	5	2
**Deprescribing**	**PPI rank**	**Non-PPI rank**
How can we empower patients to take a more active role in self-management and self-monitoring of multiple long-term conditions, including deprescribing?	1	2
Should all ‘prescribing’ guidelines include recommendations to deprescribe, and if so how?	2	9
How can GPs and non-medical prescribers be assisted in dealing with deprescribing of medicines originally prescribed by hospital consultants?	3a	9
Does providing full access to medical records enable more effective collaborative deprescribing decisions?	3b	8
How can pharmacological and holistic therapies be merged? E.g. deprescribing of antidepressants.	3c	18

The greatest similarities between the two types of participants were in the ‘patient concerns ‘category, where four of the top five questions were mutually identified as priorities.

## Discussion

To our knowledge, this is the first report of a multi-stakeholder approach to the development and prioritisation of research questions associated with medicines optimisation. Similar processes are used by the James Lind Alliance [27]. The current exercise adopted a systematic approach that incorporated the opinions of a wide range of stakeholders using participatory methods. The value of these stakeholders, rather than limiting the process to academic researchers and the literature, was illustrated by the additional 62 questions that were identified during Stage 2 as a result of including the extended stakeholder group, as well as the comparison of PPI and non-PPI priorities. Ten of the top 20 questions were identified by PPI; i.e. indicating the importance of including these stakeholders. Our results illustrate the importance of patient and public participants to inform research and guideline development in relation to medicines optimisation, as recommended by an earlier review [28]. The priorities identified by this process could be used by: research funders to inform future research funding initiatives; researchers to identify and address priorities in medicines optimisation research; PPI organisations to lobby for change and promote awareness; and health professionals to consider in terms of their medicines optimisation practice. Many of these priorities are likely to be of relevance to the international community despite being generated in the UK and reflect the aforementioned WHO global patient safety concern.

Several of the high priority questions reflected the extent to which patients’ views and experiences are considered during medication reviews. Few tools exist for eliciting patient priorities and preferences during consultations, including medication reviews [29]. As such, this service might benefit from the introduction of a common framework built on equity, confidence, and perceptions of acceptance [30], for defining and classifying patient-mediated interventions. Most of the questions associated with medication reviews related to ‘structural’ elements, i.e. what constitutes a ‘good’ review, and when is the ‘right’ time to undertake a review. The challenge for future work comes from operationalising these questions in objective, less value-laden terminology. This was also reflected with the prioritisation of questions regarding polypharmacy – for example, the nature of information and education that the patient would require to enable them to engage and have ownership of their medication management.

Polypharmacy is of increasing relevance due to the ageing population and the increasing number of people receiving multiple medicines [2]. The top priorities around polypharmacy identified in the present exercise reflect the challenge of how best to involve patients in decision making, especially in relation to medication reviews, and how primary care is best engineered to ensure their effectiveness.

In the UK, a range of non-medical health professionals can prescribe medicines for patient [31]. The growth in numbers of non-medical prescribers reflects the need to mitigate increasing demands on the NHS [32]. The ability to prescribe has the potential to enhance the roles of health care professionals and improve patient care by facilitating treatment provision in settings more accessible to patients and possibly in a more timely manner [33]. The top five research questions in the NMP category included the need to raise patient awareness of NMPs and the extent to which NMP training provides practitioners with the confidence to address complex polypharmacy and deprescribing issues [34]. It has been suggested that educational programmes for NMPs would benefit from considering how best to maintain the currency of practitioners’ knowledge [35], and that education aimed at the public may be warranted, to address concerns and limited awareness with regard to the diagnostic skills and status of NMPs [36].

The top-ranked ‘Deprescribing’ priority was “*how to empower patients to take a more active role in self-management and self-monitoring of multiple long-term conditions, including deprescribing?”*, which was were ranked ‘extremely important’ or ‘important’ by at least three-quarters of participants, indicating a convergence towards the desire for a broad culture shift i.e. consultations which reflect a more person-centered approach (a core component of medicines optimisation), whereby patient perspectives are an integral part of the decision-making process. Challenges associated with deprescribing include how it is defined, whether it is safe, and how these aspects are communicated to patients and health care professionals alike [37]. This reflects a desire to adopt a holistic approach to medicines optimisation, whereby patient perspectives and values are central to the consultation process, teams are inherently multidisciplinary, and where the general perspectives as well as complexities of individual cases can be addressed [38–40].

The PPI research infrastructure has existed for over a decade [41] and the importance of PPI in this prioritisation exercise was reinforced by the many differences between their views and those of the non-PPI participants [42]. Given that patient involvement in healthcare decision-making can lead to better affective, cognitive and health outcomes [43, 44], the value of involving patients in the entire research process is implicit in the top-ranked ‘patient concerns’, ‘*Is there a shared decision (with patients) about using each medicine*?’

The third WHO Global Patient Safety Challenge, Medication Without Harm, identified medication safety as a priority [45]. A separate consensus exercise identified the need to adopt technology to enhance medication safety, and to develop guidelines and standard operating procedures for high-risk patients, medications and contexts [46]. Within this current prioritisation exercise, it was seldom stated explicitly medication safety but was implicit in many questions. For example, ‘Patient concerns’ *‘What are the advantages and disadvantages of online pharmacy services in relation to access to medicines and safety, patient experience, out-of-pocket expenses, information provision?’* (rank 19), and ‘Deprescribing’ *‘Where are the gaps in education and safety (about deprescribing) and how these can be addressed is key to ensuring deprescribing is safe and effective?’* (rank 4). This observation may reflect the wording of the original NGT question and that the subsequent apportioning of questions across the four categories decreased the salience of ‘safety’. As such, priorities identified in the current exercise may differ substantially from global priorities where, for example, medicine safety is a recognised priority [47].

### Strengths and limitations

A strength of this prioritisation exercise was the representation at each stage of different stakeholder groups (including PPI, pharmacists, and GPs) to ensure that their different perspectives and experiences were represented throughout the process. This is likely to have improved the relevance and real-world value of the research outcomes, as well as the validity and reliability of the findings [48]. Conversely, however, the high proportion of pharmacist participants could have influenced the results although the overall findings suggest that this did not occur. If this approach was repeated with a wider range of stakeholders, e.g. nurses and other NMPs and health professionals, different priorities could have been generated. Similarly, if repeated at a different time, the outcomes could be influenced by high profile health concerns e.g. COVID-19. An additional limitation relates to the Stage 3 procedure of aggregating ratings into an overall rank. Ascribing a score to each rating and then summing the scores for a set of ratings by any one individual may produce the same magnitude of outcome from quite different sets of ratings. The Borda count employed in Stage 3 overcomes this to some degree in that it takes all the rating preferences into account by attributing weighted scores to each rating. However, this still assumes that scores are interval in nature and does not take into account the actual ‘attitude’ that the respondent may have towards the question content [49]. Nonetheless, to test whether different scoring approaches *significantly* altered the nature of rankings, different methods of analysis were explored. For example, given that ratings were skewed toward positive assessments, rankings were constructed based on aggregates of only ‘extremely important’ and ‘important’ ratings. While this did not affect overall ranks substantially, it was decided that a full Borda count that incorporated all ratings was more apposite in that it maintained the full range of perspectives from all participants.

## Conclusions

We illustrate the value of co-producing a prioritised research agenda for medicines optimisation using a multi-stakeholder approach. Hence, the results and priorities identified are relevant for clinicians, researchers, funding bodies, and policy makers, in terms of the future research agenda. Moreover, the results demonstrate the importance and value of adopting an inclusive approach with agenda-setting for health care.

## Supplementary Information

**Additional file 1.**

## Data Availability

The datasets used and/or analysed during the current exercise are available from the corresponding author on request.

## Abbreviations

NGT: Nominal group technique
NHS: National Health Service
NMP: Non-medical prescribing
PPI: Public and Patient Involvement
UK: United Kingdom
WHO: World Health Organisation
