# Supplementary material for: A multi-stakeholder approach to the co-production of the research agenda for medicines optimisation
Source: BMC Health Serv Res. 2021 Jan 13;21:64. doi: 10.1186/s12913-021-06056-5 (PMC7804576; doi:10.1186/s12913-021-06056-5)
Supplement: Supplementary file 1 — Additional file 1. [file 12913_2021_6056_MOESM1_ESM.docx]

**Additional material**

Results of the prioritisation exercise of ‘Patient concerns’ questions with rank, and percentage of participants rating questions as ‘extremely important’ or ‘important’.

Questions receiving an ‘extremely important’ or ‘important’ rating from at least three-quarters of participants are indicated with *.

Legend:

PPI = Public and Patient Involvement representative.

Non-PPI = Non-Public and Patient Involvement representative.

'Extremely important' = EI; ‘Important' = I; 'Somewhat important' = SI; Neutral' = N; ‘Somewhat unimportant' = SU; ‘Unimportant' = U; ‘Extremely important' = EU.

|  | **PPI ratings** | | | | | | | **Non-PPI ratings** | | | | | | |  | |
| --- | --- | --- | --- | --- | --- | --- | --- | --- | --- | --- | --- | --- | --- | --- | --- | --- |
| **Question** | **EI** | **I** | **SI** | **N** | **SU** | **U** | **EU** | **EI** | **I** | **SI** | **N** | **SU** | **U** | **EU** | **RANK** | **Percentage 'extremely important' + 'important'** |
| To what extent are patients’ views and experiences considered during medication reviews? | 4 | 1 | 1 | 0 | 1 | 0 | 0 | 15 | 3 | 5 | 1 | 0 | 0 | 0 | **1** | **74** |
| *Is there a shared decision (with patients) about using each medicine?* | 3 | 3 | 1 | 0 | 0 | 0 | 0 | 10 | 8 | 2 | 3 | 0 | 1 | 0 | **2** | **77** |
| How are medication reviews by different healthcare professionals integrated? | 4 | 1 | 1 | 1 | 0 | 0 | 0 | 8 | 7 | 6 | 3 | 0 | 0 | 0 | **3** | **65** |
| How can communication relevant to medication reviews be enhanced across and within sectors? | 2 | 3 | 1 | 1 | 0 | 0 | 0 | 10 | 7 | 4 | 2 | 0 | 0 | 1 | **4** | **71** |
| What information/education would patients require regarding polypharmacy to engage and take some ownership? | 1 | 4 | 1 | 1 | 0 | 0 | 0 | 8 | 9 | 4 | 1 | 1 | 1 | 0 | **5** | **71** |
| Do patients understand what each medicine is for, how/how long to take, and the choice they have? | 3 | 0 | 2 | 2 | 0 | 0 | 0 | 12 | 3 | 6 | 0 | 1 | 1 | 1 | **6** | **58** |
| During medication reviews, how can patients be encouraged to contribute to a "real picture" of what they are actually taking? | 2 | 2 | 2 | 1 | 0 | 0 | 0 | 6 | 10 | 3 | 4 | 1 | 0 | 0 | **7** | **65** |
| How are medication reviews initiated and do these reviews meet the needs of those involved? | 3 | 0 | 2 | 0 | 1 | 0 | 1 | 9 | 5 | 8 | 2 | 0 | 0 | 0 | **7** | **55** |
| What are patients’ support needs in relation to polypharmacy, and now do these vary across social groups? (Added: vary between different types of illness e.g. psychosis, dementia) | 1 | 4 | 2 | 0 | 0 | 0 | 0 | 6 | 8 | 3 | 6 | 0 | 1 | 0 | **9** | **61** |
| What are patients’ experiences of being prescribed, and of using or not using, multiple medicines, and how does this vary across social groups? (Added: vary between different types of illness e.g. psychosis, dementia) | 0 | 4 | 2 | 0 | 1 | 0 | 0 | 4 | 10 | 5 | 2 | 3 | 0 | 0 | **10** | **58** |
| Could patients be given realistic and more useful information (as opposed to PILs) re the possible long-term side effects of medication? | 3 | 2 | 1 | 0 | 0 | 0 | 1 | 2 | 11 | 4 | 5 | 1 | 1 | 0 | **11** | **58** |
| Does the frequency of medication reviews meet patient needs? | 1 | 3 | 2 | 1 | 0 | 0 | 0 | 4 | 6 | 7 | 4 | 3 | 0 | 0 | **12** | **45** |
| How do patients and carers experience deprescribing interventions, and how does this vary across social groups? (Added: vary between different types of illness e.g. psychosis, dementia) | 1 | 3 | 2 | 0 | 0 | 1 | 0 | 5 | 7 | 5 | 5 | 1 | 0 | 1 | **12** | **52** |
| What are patients’ perspectives and experiences of non-medical prescribers, and does this vary according to different professional groups and different patient groups? | 2 | 4 | 0 | 1 | 0 | 0 | 0 | 6 | 4 | 5 | 3 | 4 | 2 | 0 | **14** | **52** |
| How can systems (both digital and organisational) be used to minimise inappropriate accumulation and wastage of medicines | 3 | 0 | 3 | 0 | 0 | 1 | 0 | 4 | 6 | 3 | 5 | 5 | 1 | 0 | **15** | **42** |
| How can we avoid the accumulation of medicines (or hoarding behaviour) by patients? | 0 | 3 | 2 | 1 | 0 | 0 | 1 | 4 | 5 | 5 | 5 | 4 | 1 | 0 | **16** | **39** |
| What are patients' perspectives of non-medical prescribers in relation to their GP? | 1 | 1 | 3 | 0 | 1 | 0 | 1 | 2 | 6 | 7 | 5 | 2 | 1 | 1 | **17** | **32** |
| What would a patient do if prescribed a medicine they do not want to take? | 1 | 2 | 1 | 1 | 1 | 0 | 1 | 4 | 5 | 5 | 5 | 2 | 2 | 1 | **17** | **39** |
| How aware are patients/carers of the knowledge/skills of pharmacists? | 2 | 1 | 1 | 1 | 2 | 0 | 0 | 2 | 2 | 10 | 5 | 3 | 2 | 0 | **19** | **23** |
| What are the advantages and disadvantages of online pharmacy services in relation to access to medicines and safety, patient experience, out-of-pocket expenses, information provision? | 0 | 2 | 2 | 2 | 0 | 0 | 1 | 3 | 4 | 6 | 5 | 3 | 1 | 2 | **20** | **29** |
| How is online prescribing monitored to ensure prescribing is appropriate? | 0 | 1 | 4 | 1 | 1 | 0 | 0 | 2 | 5 | 3 | 6 | 4 | 3 | 1 | **21** | **26** |
| What range of online pharmacy services exist, what is their purpose and how and why do patients use them? | 0 | 0 | 2 | 2 | 1 | 1 | 1 | 0 | 6 | 5 | 4 | 6 | 2 | 1 | **22** | **19** |

**Table 2** Results of the prioritisation exercise prioritisation of ‘Polypharmacy’ questions with rank, and percentage of participants rating questions as ‘extremely important’ or ‘important’. Questions receiving an ‘extremely important’ or ‘important’ rating from at least three-quarters of participants are indicated with *.

|  | **PPI ratings** | | | | | | | **Non-PPI ratings** | | | | | | |  | |
| --- | --- | --- | --- | --- | --- | --- | --- | --- | --- | --- | --- | --- | --- | --- | --- | --- |
| **Question** | **EI** | **I** | **SI** | **N** | **SU** | **U** | **EU** | **EI** | **I** | **SI** | **N** | **SU** | **U** | **EU** | **RANK** | **Percentage 'extremely important' + 'important'** |
| To what extent should polypharmacy involve patients' views and preferences in shared decision making?* | 5 | 1 | 0 | 1 | 0 | 0 | 0 | 8 | 10 | 6 | 1 | 0 | 0 | 0 | **1** | **77** |
| *If we undertake a polypharmacy medication review well with the right person at the right time, what happens to the patient; ie are their outcomes better, does their QoL improve etc.?* | 4 | 2 | 0 | 0 | 0 | 0 | 1 | 10 | 9 | 4 | 1 | 1 | 0 | 0 | **2** | **81** |
| *How can we reengineer primary care to make time for medication review to be done well by the right people (based on the fact that this is only going to get bigger i.e. more older people on more drugs)?* | 1 | 5 | 0 | 0 | 1 | 0 | 0 | 10 | 10 | 3 | 1 | 0 | 1 | 0 | **3** | **84** |
| How do we achieve a "culture shift" to prevent polypharmacy (including engagement with public, addressing socioeconomic disparities)? | 2 | 1 | 2 | 1 | 1 | 0 | 0 | 14 | 2 | 7 | 2 | 0 | 0 | 0 | **4** | **61** |
| How should health services be redesigned to cope with polypharmacy/multimorbidity? | 0 | 3 | 4 | 0 | 0 | 0 | 0 | 15 | 3 | 2 | 4 | 0 | 1 | 0 | **5** | **68** |
| What are the key components of a good, person centred, holistic, polypharmacy medication review? | 4 | 1 | 1 | 0 | 1 | 0 | 0 | 10 | 5 | 7 | 2 | 1 | 0 | 0 | **6** | **65** |
| Which patients should be targeted by polypharmacy medication reviews, where and when? | 2 | 4 | 1 | 0 | 0 | 0 | 0 | 8 | 6 | 8 | 2 | 0 | 1 | 0 | **7** | **65** |
| What do clinicians need to address polypharmacy? | 2 | 2 | 3 | 0 | 0 | 0 | 0 | 7 | 7 | 6 | 3 | 2 | 0 | 0 | **8** | **58** |
| What can patients teach health care professionals about the burden of polypharmacy and coping (or not) mechanisms? | 4 | 1 | 2 | 0 | 0 | 0 | 0 | 6 | 8 | 6 | 2 | 0 | 3 | 0 | **9** | **61** |
| How can inappropriate polypharmacy be prevented? | 2 | 1 | 1 | 3 | 0 | 0 | 0 | 10 | 4 | 7 | 1 | 1 | 2 | 0 | **10** | **55** |
| How can we ensure a consistent approach to polypharmacy across health care professionals? | 3 | 2 | 1 | 0 | 1 | 0 | 0 | 4 | 9 | 7 | 3 | 0 | 2 | 0 | **11** | **58** |
| What are the professional barriers of deprescribing effectively, with problem polypharmacy? | 2 | 2 | 1 | 2 | 0 | 0 | 0 | 7 | 7 | 6 | 2 | 2 | 0 | 0 | **11** | **58** |
| Does fully involving patient/carer in prescribing decisions vs alternatives have an impact on the number of medicines prescribed? | 3 | 1 | 1 | 0 | 1 | 0 | 1 | 6 | 7 | 5 | 1 | 4 | 1 | 1 | **13** | **55** |
| What should be the intended outcomes of polypharmacy interventions? | 2 | 2 | 2 | 0 | 0 | 0 | 1 | 9 | 3 | 5 | 2 | 3 | 1 | 1 | **14** | **52** |
| What is the sustainability and efficacy of interventions to address polypharmacy? | 0 | 1 | 2 | 2 | 0 | 0 | 2 | 6 | 5 | 6 | 6 | 2 | 0 | 0 | **15** | **39** |
| Why does inappropriate polypharmacy occur? | 3 | 0 | 2 | 1 | 0 | 0 | 1 | 5 | 5 | 5 | 4 | 4 | 1 | 1 | **16** | **42** |
| Who is taking responsibility for polypharmacy reviews in multimorbidity? | 3 | 0 | 2 | 1 | 0 | 0 | 1 | 5 | 5 | 4 | 5 | 4 | 1 | 1 | **17** | **42** |
| What is the impact on society of a social movement away from lots of drugs towards greater self-care and how is that different in different populations? (Deprivation, cognitive decline etc)? | 3 | 0 | 1 | 1 | 0 | 1 | 1 | 3 | 6 | 4 | 8 | 3 | 0 | 1 | **18** | **39** |
| How should we manage polypharmacy of some conditions e.g. pain management where drugs are used because alternative non-medication services unavailable? | 1 | 2 | 2 | 2 | 0 | 0 | 0 | 2 | 4 | 7 | 4 | 6 | 1 | 1 | **19** | **29** |
| What is the role of hospital consultants in reducing polypharmacy? | 1 | 3 | 1 | 2 | 0 | 0 | 0 | 2 | 6 | 3 | 7 | 2 | 4 | 1 | **20** | **39** |
| Would holistic treatment eliminate non-effective polypharmacy? | 4 | 1 | 1 | 0 | 0 | 0 | 1 | 2 | 4 | 5 | 7 | 3 | 0 | 4 | **21** | **35** |
| How can pay for performance be used to help address inappropriate polypharmacy? | 0 | 0 | 1 | 1 | 4 | 1 | 0 | 4 | 6 | 5 | 4 | 2 | 3 | 1 | **22** | **32** |
| What combination of medications are particularly prevalent? | 1 | 3 | 2 | 0 | 0 | 1 | 0 | 1 | 3 | 5 | 9 | 2 | 4 | 1 | **23** | **26** |
| Can the life course of medication use by individuals be characterised? | 0 | 1 | 1 | 2 | 1 | 0 | 2 | 3 | 5 | 2 | 7 | 3 | 4 | 1 | **24** | **29** |
| Could an incremental approach be used with single condition guidelines and the inclusion of additional co-morbidities? | 0 | 1 | 1 | 3 | 0 | 0 | 2 | 1 | 1 | 10 | 7 | 3 | 2 | 1 | **25** | **10** |
| To what extent are polypharmacy interventions theory-based? | 0 | 0 | 1 | 2 | 0 | 1 | 3 | 2 | 3 | 4 | 4 | 5 | 3 | 3 | **27** | **16** |
| How useful is the PROMISE tool for polypharmacy interventions? | 0 | 0 | 0 | 3 | 2 | 0 | 2 | 2 | 0 | 3 | 7 | 4 | 2 | 6 | **28** | **6** |

**Table 3** Results of the prioritisation exercise prioritisation of ‘Non-medical prescribing questions with rank, and percentage of participants rating questions as ‘extremely important’ or ‘important’. Questions receiving an ‘extremely important’ or ‘important’ rating from at least three-quarters of participants are indicated with *.

|  | **PPI ratings** | | | | | | | **Non-PPI ratings** | | | | | | |  | |
| --- | --- | --- | --- | --- | --- | --- | --- | --- | --- | --- | --- | --- | --- | --- | --- | --- |
| **Question** | **EI** | **I** | **SI** | **N** | **SU** | **U** | **EU** | **EI** | **I** | **SI** | **N** | **SU** | **U** | **EU** | **RANK** | **Percentage 'extremely important' + 'important'** |
| How can we optimise the contribution of NMPs to primary care? | 4 | 1 | 2 | 0 | 0 | 0 | 0 | 12 | 6 | 5 | 2 | 0 | 0 | 0 | **1** | **74** |
| How is deprescribing, polypharmacy, etc. undertaken by NMPs? | 1 | 3 | 3 | 0 | 0 | 0 | 0 | 2 | 11 | 10 | 2 | 0 | 0 | 0 | **2** | **55** |
| What are the influences on deprescribing behaviour of NMPs? | 2 | 2 | 2 | 0 | 0 | 1 | 0 | 7 | 9 | 5 | 1 | 1 | 1 | 0 | **3** | **65** |
| To what extent does NMP training provide HCPs with the confidence to address complex polypharmacy and deprescribing issues with patients; address inappropriate polypharmacy? | 2 | 3 | 0 | 1 | 0 | 0 | 1 | 7 | 6 | 7 | 2 | 2 | 0 | 1 | **4** | **58** |
| What is the role of the NMP in medicines reviews (e.g. in specialist areas such as rheumatology and type of NMP e.g pharmacist, nurse? | 2 | 2 | 3 | 0 | 0 | 0 | 0 | 3 | 9 | 8 | 2 | 2 | 1 | 0 | **4** | **52** |
| What are the influences on the prescribing behaviour (for example the prescription of antibiotics) of NMPs? | 1 | 4 | 0 | 2 | 0 | 0 | 0 | 4 | 7 | 9 | 1 | 4 | 0 | 0 | **6** | **52** |
| How can we raise patient awareness of these ‘new’ prescribers? | 4 | 2 | 0 | 0 | 1 | 0 | 0 | 5 | 5 | 7 | 2 | 3 | 2 | 0 | **7** | **52** |
| Are different types of interventions required to change the prescribing behaviour of different groups of prescribers? | 1 | 1 | 3 | 1 | 1 | 0 | 0 | 4 | 7 | 7 | 3 | 3 | 1 | 0 | **8** | **42** |
| Are NMPs more or less likely to deprescribe medicines initiated by medical prescribers? | 0 | 1 | 2 | 3 | 0 | 0 | 1 | 5 | 8 | 8 | 1 | 0 | 2 | 1 | **9** | **45** |
| Do NMPs help to increase continuity of care? | 0 | 5 | 2 | 0 | 0 | 0 | 0 | 3 | 7 | 5 | 4 | 4 | 0 | 1 | **10** | **48** |
| How can NMPs optimise medicines use in vulnerable patient populations e.g. drug misusers, individuals with mental illness? | 1 | 4 | 2 | 0 | 0 | 0 | 0 | 0 | 4 | 12 | 6 | 1 | 1 | 0 | **11** | **29** |
| How do we best use a pharmacist NMP vs a nurse NMP etc.? | 0 | 3 | 0 | 2 | 1 | 0 | 1 | 5 | 7 | 6 | 1 | 1 | 3 | 2 | **12** | **48** |
| How do medical prescribers feel about change in culture, responsibility, workforce and skill mix to maximise potential impact of NMPs? | 1 | 2 | 1 | 1 | 1 | 0 | 1 | 2 | 7 | 7 | 4 | 3 | 1 | 0 | **13** | **39** |
| What are the minimum expectations of a health care professional conducting a medication review? | 0 | 2 | 1 | 1 | 0 | 2 | 1 | 5 | 3 | 8 | 3 | 1 | 1 | 3 | **14** | **32** |
| How to NMPs respond to medicines initiated by other NMPs? | 0 | 4 | 2 | 0 | 0 | 1 | 0 | 0 | 4 | 7 | 3 | 5 | 4 | 1 | **15** | **26** |
| Should deprescribing be carried out under the supervision of a doctor? | 1 | 2 | 2 | 0 | 0 | 1 | 1 | 2 | 1 | 8 | 6 | 2 | 1 | 5 | **16** | **19** |
| How does NMP training around ensuring on-going benefit to the patient each time a prescription is signed fit with volume of repeat prescriptions in primary care? | 2 | 0 | 3 | 1 | 1 | 0 | 0 | 1 | 3 | 4 | 5 | 5 | 2 | 3 | **17** | **19** |

**Table 4** Results of the prioritisation exercise prioritisation of ‘Deprescribing questions with rank, and percentage of participants rating questions as ‘extremely important’ or ‘important’. Questions receiving an ‘extremely important’ or ‘important’ rating from at least three-quarters of participants are indicated with *.

|  | **PPI ratings** | | | | | | | **Non-PPI ratings** | | | | | | |  | |
| --- | --- | --- | --- | --- | --- | --- | --- | --- | --- | --- | --- | --- | --- | --- | --- | --- |
| **Question** | **EI** | **I** | **SI** | **N** | **SU** | **U** | **EU** | **EI** | **I** | **SI** | **N** | **SU** | **U** | **EU** | **RANK** | **Percentage 'extremely important' + 'important'** |
| *How can we empower patients to take a more active role in self-management and self-monitoring of multiple long term conditions, including deprescribing? | 6 | 1 | 0 | 0 | 0 | 0 | 0 | 8 | 12 | 2 | 1 | 1 | 0 | 0 | **1** | **87** |
| To what extent is shared decision-making incorporated into deprescribing consultations? | 1 | 1 | 3 | 1 | 1 | 0 | 0 | 12 | 6 | 6 | 1 | 0 | 0 | 0 | **2** | **65** |
| Where are the gaps in education and training (about deprescribing) and how these can be addressed is key to ensuring deprescribing is safe and effective? | 1 | 4 | 0 | 2 | 0 | 0 | 0 | 9 | 7 | 4 | 4 | 1 | 0 | 0 | **3** | **68** |
| Should all ‘prescribing’ guidelines include recommendations to deprescribe, and if so how? | 4 | 3 | 0 | 0 | 0 | 0 | 0 | 4 | 11 | 3 | 6 | 0 | 1 | 0 | **4** | **71** |
| What should the core outcome set be for deprescribing interventions? | 1 | 3 | 1 | 2 | 0 | 0 | 0 | 8 | 9 | 2 | 4 | 2 | 0 | 0 | **5** | **68** |
| Does providing full access to medical records enable more effective collaborative deprescribing decisions? | 3 | 2 | 2 | 0 | 0 | 0 | 0 | 5 | 10 | 4 | 3 | 3 | 0 | 0 | **6** | **65** |
| How can GPs and NMPs be assisted in dealing with deprescribing of medicines originally prescribed by hospital consultants? | 3 | 3 | 0 | 1 | 0 | 0 | 0 | 6 | 10 | 3 | 3 | 2 | 0 | 0 | **7** | **71** |
| Does taking a holistic approach, rather than a combination of disease specific interventions, make a difference to polypharmacy and deprescribing? | 3 | 1 | 1 | 0 | 1 | 1 | 0 | 9 | 7 | 3 | 3 | 2 | 1 | 0 | **8** | **65** |
| What are the levers to improve engagement with deprescribing by GPs (e.g. loss of QOF incentive for meds review was a problem)? | 1 | 1 | 3 | 2 | 0 | 0 | 0 | 7 | 8 | 5 | 4 | 1 | 0 | 0 | **8** | **55** |
| Do HCPs undertake shared decision making when considering deprescribing? | 3 | 1 | 2 | 1 | 0 | 0 | 0 | 7 | 7 | 6 | 2 | 1 | 1 | 1 | **10** | **58** |
| How are healthcare professionals educated to deprescribe? | 0 | 2 | 5 | 0 | 0 | 0 | 0 | 11 | 5 | 2 | 2 | 3 | 1 | 1 | **11** | **58** |
| How do we know that deprescribing is appropriate? | 2 | 2 | 2 | 0 | 1 | 0 | 0 | 6 | 8 | 4 | 3 | 2 | 2 | 0 | **12** | **58** |
| What is patient understanding regarding deprescribing and how does this vary by medicine type, disease, stage of life? | 2 | 1 | 3 | 0 | 1 | 0 | 0 | 7 | 6 | 5 | 4 | 2 | 0 | 0 | **13** | **52** |
| What methods can be used to avoid conflict between primary and secondary care regarding deprescribing decisions, actions and maintenance? | 2 | 0 | 4 | 0 | 0 | 1 | 0 | 4 | 11 | 3 | 5 | 0 | 2 | 0 | **14** | **55** |
| To what extent do different drivers influence the outcome of deprescribing interventions? | 0 | 3 | 2 | 2 | 0 | 0 | 0 | 5 | 7 | 4 | 7 | 1 | 0 | 1 | **15** | **48** |
| How can we support patients in lay deprescribing (but support them to persist with preventive medication)? (Alternative phrasing: is there a role for lay deprescribing?) | 3 | 2 | 0 | 1 | 0 | 0 | 1 | 6 | 5 | 6 | 2 | 2 | 3 | 1 | **16** | **52** |
| How can pharmacological and holistic therapies be merged? e.g. deprescribing of antidepressants | 3 | 2 | 2 | 0 | 0 | 0 | 0 | 4 | 4 | 4 | 7 | 4 | 1 | 1 | **17** | **42** |
| What are the barriers to deprescribing during hospital admissions? | 1 | 3 | 1 | 2 | 0 | 0 | 0 | 4 | 6 | 5 | 1 | 7 | 1 | 1 | **18** | **45** |
| What are the ethical barriers to deprescribing? | 0 | 1 | 3 | 2 | 1 | 0 | 0 | 3 | 5 | 7 | 4 | 3 | 1 | 0 | **19** | **29** |
| What definition of deprescribing should be used which encompasses patient (all stakeholders’) opinion? | 1 | 3 | 1 | 1 | 0 | 1 | 0 | 2 | 5 | 6 | 2 | 5 | 1 | 4 | **20** | **35** |
| GPs need to be a key target - partly because they do the prescribing, partly because some still are unconvinced about the risks it poses and are also concerned about the potential dangers of deprescribing? | 2 | 2 | 0 | 1 | 1 | 0 | 1 | 3 | 5 | 4 | 3 | 3 | 2 | 5 | **21** | **39** |
| Is deprescribing taught in undergraduate curriculum? | 2 | 2 | 0 | 0 | 0 | 1 | 2 | 1 | 4 | 6 | 5 | 6 | 1 | 2 | **22** | **29** |
| Which deprescribing guidelines are available? | 0 | 2 | 3 | 1 | 0 | 0 | 1 | 1 | 3 | 7 | 5 | 3 | 1 | 5 | **23** | **19** |
| To what extent is deprescribing driven by CCG and GP budgetary constraints? | 1 | 1 | 1 | 2 | 1 | 0 | 1 | 1 | 5 | 4 | 5 | 2 | 4 | 4 | **24** | **26** |
| What do the general public think about the term deprescribing and should we use it? | 0 | 1 | 3 | 1 | 1 | 0 | 1 | 1 | 7 | 2 | 4 | 2 | 4 | 4 | **25** | **29** |
